# Dramatic response to crizotinib through MET phosphorylation inhibition in rare TFG-MET fusion advanced squamous cell lung cancer

**DOI:** 10.1093/oncolo/oyae166

**Published:** 2024-07-02

**Authors:** Wanwan Cheng, Ting Xu, Lu Yang, Naimeng Yan, Jie Yang, Shencun Fang

**Affiliations:** Department of Respiratory Medicine, Nanjing Chest Hospital, The Affiliated Brain Hospital of Nanjing Medical University, Nanjing, China; Department of Respiratory Medicine, Nanjing Chest Hospital, The Affiliated Brain Hospital of Nanjing Medical University, Nanjing, China; The Genetic Analysis Department, YuceBio Technology Co., Ltd., Shenzhen, China; The Genetic Analysis Department, YuceBio Technology Co., Ltd., Shenzhen, China; The Genetic Analysis Department, YuceBio Technology Co., Ltd., Shenzhen, China; Department of Respiratory Medicine, Nanjing Chest Hospital, The Affiliated Brain Hospital of Nanjing Medical University, Nanjing, China

**Keywords:** NSCLC, MET rearrangement, biomarker, crizotinib, sensitive

## Abstract

With the widespread use of next-generation sequencing (NGS) for solid tumors, mesenchymal-to-epithelial transition factor (MET) rearrangement/fusion has been confirmed in multiple cancer types. MET amplification and MET exon 14 skipping mutations induce protein autophosphorylation; however, the pathogenic mechanism and drug sensitivity of MET fusion remain unclear. The following report describes the clinical case of a patient diagnosed with squamous lung cancer bearing a TFG-MET gene fusion. In vitro assays demonstrated MET phosphorylation and oncogenic capacity due to the TFG-MET rearrangement, both of which were inhibited by crizotinib treatment. The patient was treated with crizotinib, which resulted in sustained partial remission for more than 17 months. Collectively, cellular analyses and our case report emphasize the potential of MET fusion as a predictive biomarker for personalized target therapy for solid tumors.

## Teaser

The following report describes the clinical case of a patient diagnosed with squamous lung cancer bearing a TFG-MET gene fusion, emphasizing the potential of MET fusion as a predictive biomarker for personalized target therapy for solid tumors.

**Key points**

- Crizotinib treatment showed a favorable response in a patient with advanced squamous cell lung cancer harboring the TFG-MET fusion gene.
- The TFG-MET fusion gene is carcinogenic due to phosphorylation, which was inhibited by crizotinib, as evidenced in Ba/F3 cells.
- MET fusion may be a tumor-agnostic biomarker guiding precision oncology treatment of pan-solid tumors, such as MSI, NTRK, TMB, and BRAF-V600E.

## Introduction

The mesenchymal epithelial transition factor (MET) gene is a proto-oncogene that encodes a receptor tyrosine kinase with pleiotropic functions in malignant tumors.^1^

In contrast to MET exon 14 skipping mutations and amplifications, which have been identified as common oncogenic drivers and serve as therapeutic targets in non-small cell lung cancer (NSCLC),^2^ MET rearrangements are rare in NSCLC (0.29%), and clinical management of these patients is challenging.^3^ In isolated cases, MET fusion/rearrangements have been reported to be responsive to MET inhibition, despite the lack of mechanistic information.^4-6^ MET rearrangement/fusions have been found across 10 cancer types in China.^7^ These features further highlight the importance of studying the spectrum and the oncogenic role of MET fusion as well as its targeted inhibition in solid tumors.

Herein, we report a case of lung squamous cell carcinoma (LUSC) carrying a rare TFG-MET rearrangement, which has been previously reported in other tumors but without clinical management. Our patient showed a durable response to crizotinib for 17 months. We further verified the oncogenic addiction and sensitivity of the TFG-MET to crizotinib by cellular study and explored the mechanisms by which TFG-MET drives tumorigenesis and the rationale for its susceptibility to MET inhibition.

## Patient story

**Figure 1.**
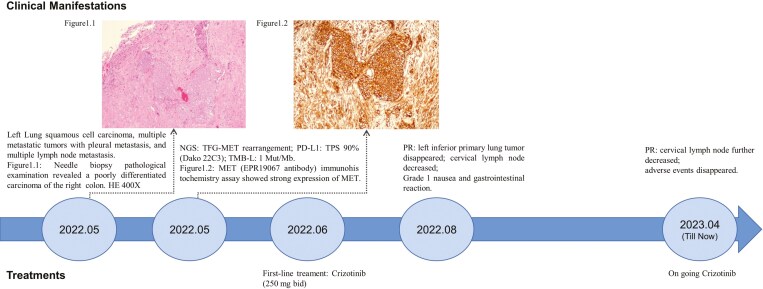
Schematic diagram showing treatment record of the patient.

In May 2022, a 70-year-old woman was admitted to the hospital with a cough. Chest computed tomography (CT) scan revealed a 2.6-cm primary mass in the inferior lobe of the left lung with, and whole body scans indicated multiple metastatic tumors including pleural metastasis, lymph node, and muscle metastases. Subsequent immunohistochemical (IHC) analysis was negative for expression of thyroid transcription factor-1 (TTF-1) and napsinA. The final diagnosis was stage IV squamous cell lung carcinoma (Figure 1).

## Molecular tumor board

**Figure 2.**
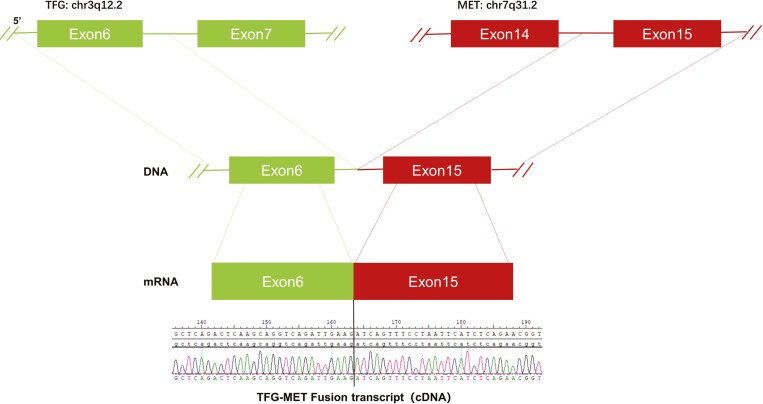
Next-generation sequencing findings for the tumor tissue sample. Diagrams showed the DNA seq around the breaking point of TFG-MET rearrangement.

A rare *TFG-MET* (exon 6: exon 15) rearrangement was identified in a metastatic right cervical lymph node using DNA-based next-generation sequencing (DNA-seq) via a customized panel including 575 genes. The TFG-MET rearrangement had breakpoints in TFG exon 6 and MET exon 15, retaining the complete regulatory dimerization domain of TFG and the intact kinase domain of MET, which could produce mature TFG-MET fusion transcripts (Figure 2). To determine the expression status of c-MET protein, we performed IHC and found positive c-MET expression (Figure 2). Other biomarkers showed low tumor mutation burden (TMB-L: 1 Muts/Mb) and low microsatellite instability (MSI-L). IHC staining was strongly positive for PD-L1 (Dako 22C3;TPS: 90%).

The National Comprehensive Cancer Network recommends immune checkpoint inhibitors (ICI) as first-line treatment for NSCLC that is negative for actionable molecular biomarkers. While not included in the usual list of actionable mutation in NSCLC, we considered that the TFG-MET fusion could be a rare actionable variant that was the single potential driver mutation in this case. This conclusion was supported by previous studies that have suggested that crizotinib can be useful in enhancing anticancer chemotherapy effects and subsequent responses to ICIs.^8,9^ According to discussion at a multidisciplinary molecular tumor board, the patient was treated with crizotinib (250 mg bid) instead of ICIs as the first-line treatment starting in June 2022.

## Patient update

**Figure 3.**
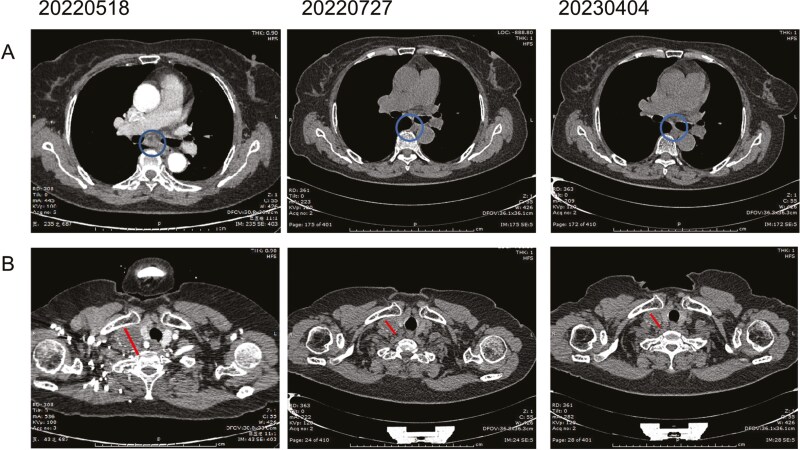
CT scans illustrating the changes in primary focal of left lung and metastatic lymph nodes over time. (A) Primary lesion at the left lung. (B) Right supraclavicular lymph node: 20220518:3.2 × 2.7 cm, 20220727:2.5 × 2.1cm, 20230404:2.3 × 1.9 cm.

Two months after crizotinib treatment, a chest CT scan showed total regression of the left inferior primary lung tumor, in combination with regression of the pleural effusion and the partial regression of the cervical lymph node (Figure 3), which indicated a partial response (PR) according to the Response Evaluation Criteria in Solid Tumors (RECIST 1.1) guidelines. The patient has received crizotinib for 17 months and maintained a durable response. Only grade 1 nausea and grade 1 gastrointestinal reaction were observed. The patient was fully compliant owing to the effective clinical response.

## Discussion

Rearrangement, a complex form of variation, is challenging during clinical management owing to the limitations of DNA detection technology and the unknown nature of the final transcription product of rearrangement at the DNA level.^10^ Existing evidence suggests that different fusion partners and fusion directions can mediate different downstream signaling pathways and may exhibit different sensitivities to tyrosine kinase inhibitors (TKIs).^11,12^ Therefore, it is important to understand the oncogenic mechanisms and drug sensitivity of MET fusions to develop precision medicine.

The protein coding gene, trafficking from the endoplasmic reticulum (ER) to Golgi regulator (TFG), is located at chr3q12.2, and is involved in the normal function of the endoplasmic reticulum and its associated microtubules.^13^ TFG contains promoter regions that stimulate the activity of downstream partner gene kinases, forming fusion genes, such as TFG-MET, TFG-ROS1, TFG-NTRK1, and TFG-ALK, to promote the development of tumors.^14^

TFG-MET fusion has been identified in different tumor types, such as thyroid cancer, glioblastoma, and spindle cell sarcoma.^15^ Although the therapeutic susceptibility of the TFG-MET fusion has not yet been reported, our study provides clinical evidence for the efficacy of crizotinib in TFG-MET rearranged LUSC. Our in vitro cell experiments demonstrated the carcinogenic effect of the TFG-MET fusion gene by increasing colony formation, cell growth, and MET phosphorylation, which was inhibited by crizotinib.

**Figure 4.**
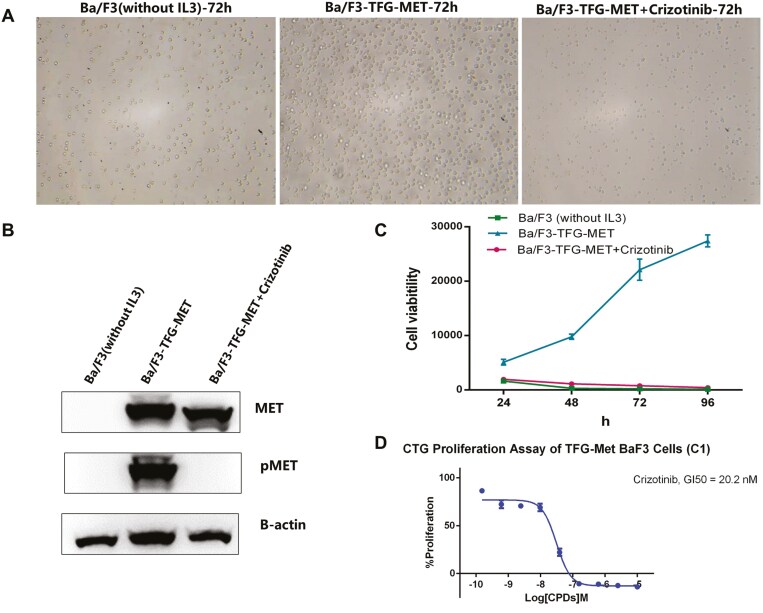
Exploration of oncogenicity and crizotinib sensitivity of TFG-MET. TFG-MET overexpressed Ba/F3 cells showed interleukin-3-independent growth whereas the Ba/F3 cells with empty vector did not. The activation could be inhibited by crizotinib (A, C). Western blot of total and phosphorylated MET of transfected Ba/F3 cells. Expression of the protein MET was detected in Ba/F3 cells with exogenous expression of the TFG-MET cDNA, and the phosphorylation of TMET could be suppressed by crizotinib. pMET, Phosphorylation of MET receptor tyrosine kinase (B). CTG result of TFG-Met BaF3 cells (C1) treated with crizotinib (D).

To understand the contribution of the TFG-MET fusion to carcinogenicity and drug susceptibility, plasmids that exogenously expressed TFG-MET fusion protein were first transduced into Ba/F3 cells. The oncogenic ability of the TFG-MET fusion gene was further validated by increased colony formation, and cell growth, which were not observed in BA/F3-without TFG-MET (Figure 4A). Second, immunoblot experiments showed that Ba/F3 cells with TFG-MET fusion demonstrated strong MET phosphorylation, which was inhibited by crizotinib (Figure 4B). This is consistent with observations in crizotinib-targeted MET amplification and MET exon14 skipping mutation, suggesting that autophosphorylation by TFG-MET fusion is an intrinsic mechanism underlying carcinogenesis and crizotinib may be effective against MET fusion. Finally, crizotinib inhibited the proliferation of Ba/F3 cells harboring the TFG-MET fusion (Figure 4A). The suppression ratio of Ba/F3-TFG-MET with crizotinib compared to Ba/F3-TFG-MET without crizotinib decreased by: 61.8%, 88.3%, 96.5%, and 98.4% on days 1, 2, 3, and 4, respectively (*P* < 0.0001) (Figure 4C). A previous study has shown that the PB1 domain in the TFG fragment plays a key role in regulating the oligomerization and activation of RET kinase, and PB1-domain-mediated oligomerization contributes to the self-phosphorylation and/or activation of RET kinase, which induces the enhancement of the TFG-RET (exon 4:exon 11) fusion carcinogenicity.^16^ Consistent with a conclusion that the PB1 domain and MET intact kinase domain are still preserved in TFG-MET (exon 6:exon 15), our cell experiments have shown that the TFG-MET fusion leads to enhanced MET phosphorylation. We speculate that PB1 domain-mediated TFG-MET oligomerization contributes to the self-phosphorylation and/or activation of MET kinase. Thus, autophosphorylation by TFG-MET fusion might be an intrinsic mechanism underlying carcinogenesis. Further, crizotinib, as a type 1A MET-TKI, can inhibit the self-phosphorylation of c-MET, being potentially effective against MET fusion.

The IC_50_ value of crizotinib for TFG-MET fusion is 20.01 nM (Figure 4D), which is similar to that for MET exon14 skipping mutation (22-28.9 nM).^17^ The median progression-free survival (PFS) was 7.3 months in patients with NSCLC harboring the MET exon 14 skipping mutation and were administered crizotinib as the first-line treatment. The PFS is comparable between a case of lung adenocarcinoma (4.0-14 months) with primary MET fusion^3^ and our case (17 months). Based on the clinical efficacy data and in vitro drug sensitivity data, we speculate that the effectiveness of crizotinib against MET fusion is parallel to that for the MET exon 14 skipping mutation, and may even be more superior in NSCLC.

MET fusions have been identified in pan-solid tumors but are rarely observed in other tumors, except for hepatocellular carcinoma (~4%) and glioma (~12%).^18,19^ Several case reports have suggested that MET TKIs benefit patients with lung cancer, hepatocellular carcinoma, glioma and other refractory tumors with MET fusion.^4,5,20,21^ However, information on the molecular mechanisms and drug susceptibility of the MET fusion in vitro and in vivo is lacking. Our study provides mechanistic and valuable evidence that TFG-MET fusion may be highly sensitive to crizotinib in Ba/F3 cells through the inhibition of MET phosphorylation. This study complements our understanding of MET fusion and further supports the hypothesis that patients with malignant tumors harboring MET fusion should have the opportunity to undergo MET-TKI therapy. Taken together, this work supports the concept that MET fusion may be another biomarker in guiding precision treatment of pan-solid tumors, such as MSI, NTRK, TMB, and BRAF-V600E.

Understanding the mechanisms of acquired resistance to crizotinib is critical to future management of our patient. Kang et al. reported that the second-site MET mutations such as D1228H/N or D1246N might be acquired resistance in EPHB4-MET, which could be suppressing effectively by tivantinib in a patient-derived organoid model.^22^ It is widely accepted that second-site MET mutations acquire resistance by MET exon 14 skipping mutation or amplification, and can be sensitive to different MET-TKIs,^9,23^ which could provide choices to treat patients with tumors bearing MET fusions that develop resistance. Whether the other known resistance mechanisms of crizotinib such as bypassing signaling pathways activation (ERBB2 amplification, KRAS mutation, etc.)^24^ are recurrent in MET fusion-resistance warrants further study.

Our study was limited by the lack of large-scale data and long-term follow-up data to truly understand the durability of the response. For subsequent studies, large-scale data and a longer follow-up would help obtain a deeper understanding of the therapeutic response to MET fusion.

Our study highlights that TFG-MET fusion may serve as a predictive biomarker for crizotinib treatment, which may provide a new perspective to guide a personalized medical approach for tumors with MET fusion. MET fusion is a potential biomarker for guiding precision oncology treatment of solid tumors. Clinical trials are needed to develop treatment strategies for solid tumors harboring MET fusion, and prospective or clinical studies are required to support these preliminary findings.

## Data Availability

The data underlying this article will be shared on reasonable request to the corresponding author.
